# Analysis of Chromosomal Numbers, Mitochondrial Genome, and Full-Length Transcriptome of *Onychostoma brevibarba*

**DOI:** 10.1007/s10126-019-09899-6

**Published:** 2019-06-15

**Authors:** Fangzhou Hu, Jingjing Fan, Chang Wu, Ming Zhu, Yunfan Zhou, Shi Wang, Chun Zhang, Min Tao, Rurong Zhao, Chenchen Tang, Kaikun Luo, Qinbo Qin, Ming Ma, Bo Chen, Jinpu Wang, Aiguo Zhou, Liangxiong Bai, Shaojun Liu

**Affiliations:** 1 State Key Laboratory of Developmental Biology of Freshwater Fish, College of Life Sciences, Hunan Normal University, Changsha, 410081 Hunan People’s Republic of China; 2 Key Laboratory of Phytochemical R&D of Hunan Province, Key Laboratory of Chemical Biology & Traditional Chinese Medicine Research, Ministry of Education, Hunan Normal University, Changsha, 410081 China; 3 Hunan Province Livestock and Fisheries Bureau, Changsha, 410081 China; 4 Chenzhou Animal Husbandry-Veterinary & Fisheries Bureau, Changsha, 410081 China; 5 Mangshan Forestry Resources Administration Bureau, Changsha, 410081 China

**Keywords:** *Onychostoma brevibarba*, Mitochondrial DNA, Chromosome and karyotype, Transcriptome, PacBio sequencing

## Abstract

*Onychostoma brevibarba* is a new discovered species which is distributed in Xiang Jiang River of the middle Chang Jiang basin in Hunan Province, South China. In this study, the ploidy levels of *O. brevibarba* were confirmed by counting chromosomal numbers and analyzing karyotype. The complete mitochondrial genome of *O. brevibarba* was determined and analyzed. Besides, we firstly performed the full-length transcriptome of *O. brevibarba* derived from 5 different tissues using the PacBio SMRT sequencing. The result shows that *O. brevibarba* was a diploid with 48 chromosomes. The complete mitogenome of *O. brevibarba* was 16,602 bp in size and very similar (89.1–91.3%) to that of other *Onychostoma* species but was distinct from all congeners. The full-length transcriptome dataset of *O. brevibarba* comprised 120,239 unigenes. Among the unigenes, 91,542 were functionally annotated, whereas 26,794 were found to have two or more isoforms. This study could provide many new insights into cytology and molecular characteristics of *O. brevibarba*; it laid the foundation for further exploration of the genomic signatures of species of *Onychostoma*.

**Electronic supplementary material:**

The online version of this article (10.1007/s10126-019-09899-6) contains supplementary material, which is available to authorized users.

## Introduction

Species in the genus *Onychostoma* are small and medium-sized freshwater fishes that belong to the order *Cypriniformes* (Cyprinidae: Barbinae) that are found throughout hill streams and in middle-and upper-river habitats (Yue et al. 2000). *Onychostoma* contains approximately 19 species (subspecies) that are distributed widely in East and Southeast Asia, and some are of high economic value (Han et al. 2015). The restriction of the lower lip, which bears a sharp, cornified sheath on the cutting edge, to the sides of the lower jaw in *Onychostoma* is almost diagnostic (Yue et al. 2000). Most of the current researches on the *Onychostoma* is about morphological characteristics and taxonomy, and the research on the cytology and molecular is rare. Only a few research involved chromosomal numbers, mitochondrial genome, and 5S rDNA (Han et al. 2015; Yu et al. 1987; Dai et al. 2012; Pei-Zhen 2012; Dai 2013; Yin and Dai 2014; Tseng et al. 2017). The research and evaluation of the genetic diversity of the species of *Onychostoma* have important practical guiding significance for the conservation and sustainable use of the fish resources.

At present, RNA-Seq has been widely used in fish genetic studies (Xu et al. 2015; Hu et al. 2017; Tao et al. 2013; Bar et al. 2016; Sharma et al. 2014). However, the differences in transcript abundance and the presence of different isoforms greatly challenge the assembly of a transcriptome from short reads. Single-molecule long-read sequencing technology from Pacific Biosciences (PacBio) (Rhoads and Au 2015; Weirather et al. 2017) has provided an efficient approach to sequence full-length cDNA molecules, which has been successively used for whole-transcriptome profiling in some species (Zhang et al. 2018a, b; Yang et al. 2018), including fishes (Yi et al. 2018).

*Onychostoma brevibarba* is a new discovered species and distributed in Xiang Jiang River of the middle Chang Jiang basin in Hunan Province, South China (Song et al. 2018). However, ploidy levels and genomic signatures about the species remain unknown. In this study, we firstly reported the chromosomal numbers, karyotype, and complete mitochondrial genome of *O. brevibarba.* Moreover, we reported the first PacBio transcriptome sequencing of *O. brevibarba*. Our result provides many new insights into cytology and molecular characteristics of *O. brevibarba*; it established the base of further research, utilization, and protection of this species.

## Materials and Methods

### Ethics Statement

Fish treatments were carried out according to the Care and Use of Agricultural Animals in Agricultural Research and Teaching, approved by the Science and Technology Bureau of China. Approval from the Department of Wildlife Administration is not required for the experiments conducted in this paper. Fish was deeply anesthetized with 100 mg/L MS-222 (Sigma-Aldrich) before dissection.

### Animal Material

**Fig. 1:**
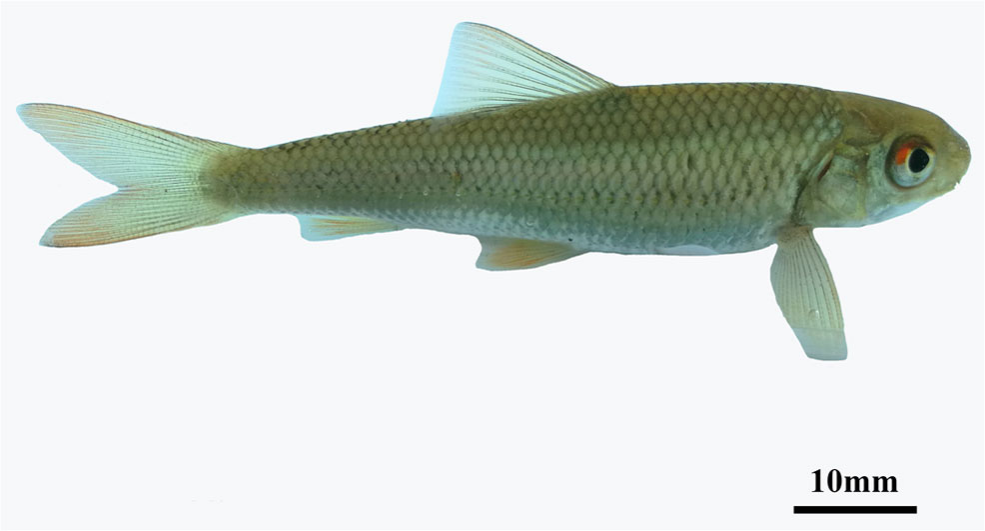
The appearance of *Onychostoma brevibarba*

The water system of Mang Mountain is upstream of the Beijiang River, which belongs to the source of the Pearl River. The *O. brevibarba* (Fig. 1) were collected from streams of two different sites between 300 and 1000 m on Mang Mountain.

### Chromosomal Numbers and Karyotype

Ten *O. brevibarba* individuals were randomly sampled for examination of chromosomal numbers and karyotype. Mitotic chromosomes were obtained from peripheral blood cell cultures following the procedures described in Xiao et al. (Xiao et al. 2014). First, about 0.1 mL blood was collected from each sample using a syringe soaked with 0.1% sodium heparin and cultured in nutrient solution at 25.5 °C and 5% CO_2_ for 72 h; then, colchicine was added 3.5 h before harvest. Cells were harvested by centrifugation, followed by hypotonic treatment with 0.075 M KCl at 26 °C for 30 min, then fixed in methanol–acetic acid (3:1, *v*/*v*) with three changes. Cells were dropped onto cold slides, air dried, and stained for 30 min in 4% Giemsa solution. Fifty metaphase spreads from ten individuals were analyzed to confirm the chromosomal numbers and the karyotype structure. The chromosomes were classified as metacentric (m), submetacentric (sm), subtelocentric (st), and telocentric (t), according to Levan et al. (Levan et al. 1964).

### Mitogenome Analyses

Two *O. brevibarba* individuals were randomly sampled for mitogenome analyses. The genomic DNA was extracted from the blood cells of *O. brevibarba* via a phenol/chloroform extraction method and used as templates. Ten pairs of polymerase chain reaction (PCR) primers (Supplementary Table 1) were designed to amplify the entire mitogenome sequence based on the conserved sequences of Cyprinid species retrieved from GenBank using Primer 5.0 software. The amplifications were performed in a 25 μL reaction volume containing 1× LA PCR buffer II (Mg^2+^), 1.25 mM of dNTPs, 0.5 mM of each primer, 1.25 Unit of LA Taq polymerase (Takara, Dalian, China), and approximately 100 ng of template genomic DNA. PCR was performed under the following conditions: denaturation at 95 °C for 5 min followed by 30 cycles at 98 °C for 10 s, 52–58 °C for 45 s, and 72 °C for 1–5 min as well as further incubation for 10 min at 72 °C. Subsequently, the targeted fragments were purified using a gel extraction kit (Sangon, Shanghai, China) and directly sequenced by the Sangon Biotechnology Company (Sangon, Shanghai, China). The sequences homology and variation among the fragments amplified from this new species were analyzed using BioEdit (Hall 1999), ClustalW (Larkin et al. 2007), and MEGA7 (Kumar et al. 2016). The published sequences of complete mtDNA were obtained from the NCBI GenBank. The accession numbers were *O. simum* (KF021233), *O. barbatulum* (KC896762), *O. barbatum* (KT438512), *O. alticorpus* (NC_021473), *O. rara* (KF626377), *O. gerlachi* (KP244449), *O. macrolepis* (KF999680), and *O. lini* (JQ343982). The phylogenetic tree was constructed on the complete sequences of mtDNA using neighbor-joining (NJ) program of MEGA (7.0) software package based on the Kimura 2-parameter model. The statistical reliability was tested using bootstrap support (BS). The BS values of nodes of the subtree were obtained after 1000 replicates. Molecular phylogenies were used primarily to support the placement of the newly collected specimens within the genus *Onychostoma*, rather than to resolve phylogenetic relationships within the group.

### RNA Extraction, Library Construction, and PacBio Sequencing

Total RNA was isolated from 5 tissues (liver, eye, muscular, kidney, and fin ray) using the TRIzol Reagent (Invitrogen) following the manufacturer’s protocol. RNA quality and concentration were determined using gel electrophoresis and Agilent Bioanalyser 2100 (Agilent Technologies, CA, USA), respectively. Equal amounts of the total RNA from each of the 5 tissues were pooled to generate one sample for library preparation. The first-strand cDNA was synthesized using SMARTer PCR cDNA Synthesis Kit (Clontech, CA, USA). After a round of PCR amplification, the amplified cDNA was size selected into different size fractions to prevent preferential small template sequencing, using the Blue Pippin (Sage Science; MA, USA). One library (0.5–6 kb) was constructed and sequenced on two cells with PacBio Sequel system (PacBio, CA, USA).

### Data Analysis of PacBio Sequencing Reads

Raw PacBio polymerase reads that have subreads ≥ 50 and a predicted consensus accuracy ≥ 0.75 were selected to produce reads of insert (ROIs), including full-length (FL) and non-full-length (nFL) transcript sequences according to whether 5′/3′ cDNA primers and a poly (A) tail were simultaneously observed. The FL sequences were subjected to isoform-level clustering by the Iso-Seq iterative clustering for error correction (ICE) algorithm to generate consensus sequences. The redundancy of the consensus sequences was removed using CD-HIT (version 4.6) to obtain isoform-level sequences. Finally, the isoform-level sequences were further clustered by CD-HIT to generate the unigene sequences.

### Gene Annotation

Functional annotations were conducted by using BLAST toolkit (*E* value ≤ 1 × 10^−5^) against different protein and nucleotide databases of Clusters of Orthologous Groups (COG); a manually annotated, non-redundant protein database (Swiss-Prot); NCBI non-redundant proteins database (NR); and Kyoto Encyclopedia of Genes Genomes (KEGG). Meanwhile, we used InterProScan5 (Philip et al. 2014) to obtain the InterPro annotation. Blast2GO (Conesa and Gotz 2008) was used to annotate with Gene Ontology (GO) based on the NR annotation.

Alternative splicing (AS) types cannot be identified due to the lack of genomic information of *O. brevibarba.* But, the corrected PacBio reads derived from a single RNA molecule, and thus, this data can be sufficient to detect gene splice site (Tilgner et al. 2013). Based on the AS gene identified for *Danio rerio*, *Takifugu rubripes*, *Oryzias latipes*, and *Gasterosteus aculeatus* (Lu et al. 2010), the potential level of conservation of annotated AS transcripts between the *O. brevibarba* and these four teleosts was conducted.

### RT-PCR Validation of AS

For PCR validation of AS, gene-specific primers were designed using Primer Premier (Version 6.0). All primers used in the RT-PCR analyses are reported in Supplementary Table 2. RNA samples were used as templates for reverse transcription with the PrimeScript RT reagent Kit (TaKara, Dalian, China). The RT-PCR was performed with an amplification protocol included initial denaturation at 95 °C for 10 min; 30–35 cycles of 95 °C for 15 s, 52–56 °C for 30 s, and 72 °C for 30 s; and 72 °C for 1 min.

#### Data Availability

All relevant supplementary data is provided within this manuscript as Supplementary files. We deposited the raw bam files in the Sequence Read Archives (SRA) of the National Center for Biotechnology Information (NCBI) under SRA accession SRR8786412.

## Results

### Chromosomal Numbers and Karyotype Analyses

**Fig. 2:**
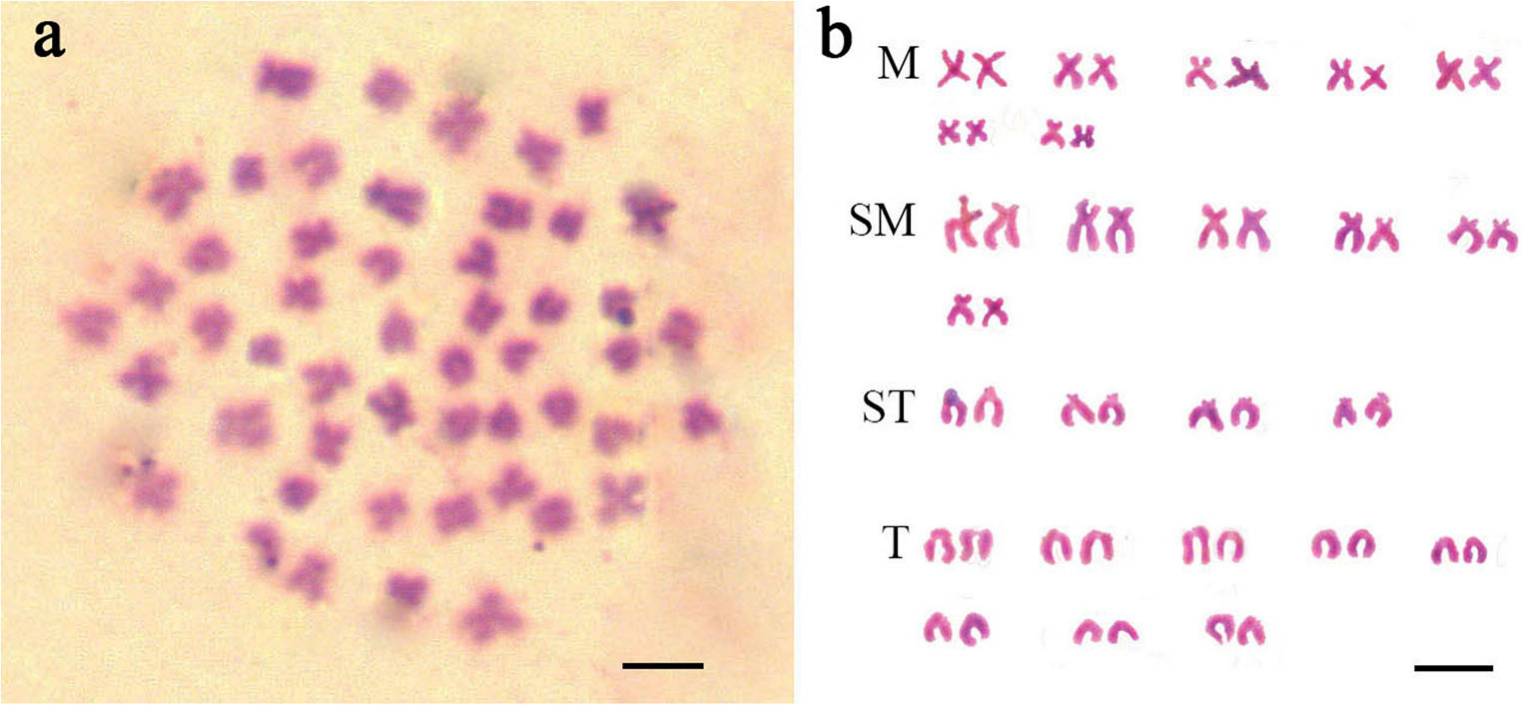
The chromosomal numbers and karyotype of *Onychostoma brevibarba.***a** The 50 chromosomes of *O. brevibarba*. **b** The karyotype of *O. brevibarba*. The scale bar in **a** and **b** represents 3 μm

Chromosomes were counted in ten metaphase spreads of *O. brevibarba*. Of the individuals examined, 95% of chromosomal metaphases possessed 50 chromosomes, indicating that they are diploids with 50 chromosomes (2*n* = 50) (Fig. 2a). The karyotype analysis indicated that the karyotype is 14 m + 12 sm + 8 st + 16 t (Fig. 2b and Table 1).

**Table 1:** Chromosomal numbers and karyotypic formulae of different *Onychostoma* species

Species	Chromosome number	Karyotypic formula
*O. brevibarba*	50	14 m + 12 sm + 10 st + 6 t
*O. simus*	50	10 m + 16 sm + 16 st + 8 t
*O. rara*	50	12 m + 16 sm + 10 st + 12 t
*O. lini*	50	12 m + 8 sm + 4 st + 26 t
*O. gerlachi*	50	12 m + 12 sm + 14 st + 12 t
*O. barbatulum*	50	10 m + 24 sm + 16 a (♀)
		10 m + 23 sm + 17 a (♂)
*O. alticorpus*	50	6 m + 26 sm + 18 a (♀)
		6 m + 27 sm + 17 a (♂)
*O. macrolepis*	50	16 m + 14 sm + 8 t

### Mitogenome and Phylogenetic Analyses

The complete mitogenome of *O. brevibarba* was 16,602 bp in size with 13 protein-coding genes (PCGs), 22 transfer RNA (tRNA) genes, and a control region (CR) (GenBank accession: MG523272). Besides, the 1550 bp COI gene sequence was identified among the nine individuals. Most of the mitochondrial genes were encoded on a heavy strand (H-strand), except for NADH dehydrogenase subunit 6 (ND6) and eight tRNA genes (tRNA-Gln, tRNA-Ala, tRNA-Asn, tRNA-Cys, tRNA-Tyr, tRNA-Ser1, tRNA-Glu, and tRNA-Pro), which were encoded on a light strand (OL). The gene composition, arrangement, and transcriptional orientation in *O. brevibarba* are similar to that of other vertebrates (Table 2). The overall base composition of the H-strand was 31.38% A, 25.10% T, 27.44% C, and 16.08% G (Table 3). All PCGs started with the standard ATG codon, except the COI gene which used GTG as the starting codon. TAA was the typical stop codon in six PCGs (ND1, COI, ATP6, COIII, ND4L, and ND5) while TAG was the stop codon for four PCGs (ND2, ATP8, ND3, and ND6), and the other three genes (COII, ND4 and Cytb) had an incomplete T stop codon. The 12S ribosomal RNA (rRNA) (959 bp) and 16S rRNA (1703 bp) genes were separated by the tRNA-Val gene. Twenty-two tRNA genes ranged from 67 bp (tRNA-Cys) to 77 bp (tRNA-Glu). All tRNAs exhibited a typical clover-leaf secondary structure, except tRNA-Ser2, which lacked the dihydrouridine arm and was replaced with a simple loop. Between tRNA-Asn and tRNA-Cys, a 47-bp sequence was identified as the origin of the replication of the L-strand (OL), which could be folded into a hairpin structure. The A- and T-rich (65.68%) CR (944 bp) were located between tRNA-Pro and tRNA-Phe (Table 2).

**Table 2:** Characteristics of the mitochondrial genome of *O. brevibarba*

Gene	Start position	Stop position	Start codon	Stop codon	Size (bp)	Strand (sense)
*tRNA-Phe*	1	69			69	H
*12sRNA*	70	1028			959	H
*tRNA-Val*	1026	1097			72	H
*16sRNA*	1097	2799			1703	H
*tRNA-Leu*	2780	2855			76	H
*ND1*	2856	3830	ATG	TAG	975	H
*tRNA-Ile*	3837	3908			72	H
*tRNA-Gln*	3907	3977			71	L
*tRNA-Met*	3981	4049			69	H
*ND2*	4050	5096	ATG	TAG	1047	H
*tRNA-trp*	5095	5165			71	H
*tRNA-Ala*	5168	5236			69	L
*tRNA-Asn*	5238	5310			73	L
*O*l	5307	5353			47	
*tRNA-Cys*	5344	5410			67	L
*tRNA-Tyr*	5410	5480			71	L
*C*oI	5482	7032	GTG	TAA	1551	H
*tRNA-Ser*	7033	7103			71	L
*tRNA-Asp*	7106	7178			73	H
*C*oII	7192	7882	ATG	T--	691	H
*tRNA-Lys*	7883	7958			76	H
*A*TP8	7960	8124	ATG	TAA	165	H
*A*TP6	8118	8801	ATG	TAA	684	H
*CoIII*	8801	9586	ATG	TAA	786	H
*tRNA-Gly*	9586	9658			73	H
*N*D3	9659	10,009	ATG	TAG	351	H
*tRNA-Arg*	10,008	10,078			71	H
*N*D4L	10,078	10,374	ATG	TAA	297	H
*N*D4	10,368	11,747	ATG	T--	1380	H
*tRNA-His*	11,748	11,816			69	H
*tRNA-Ser*	11,817	11,885			69	H
*tRNA-Leu*	11,892	11,964			73	H
*N*D5	11,961	13,784	ATG	TAA	1824	H
*N*D6	13,781	14,302	ATG	T--	522	L
*tRNA-Gln*	14,303	14,379			77	L
*C*ytb	14,377	15,517	ATG	T--	1141	H
*tRNA-Thr*	15,518	15,589			72	H
*tRNA-Pro*	15,589	15,658			70	L
*D*-loop	15,659	16,602			944

**Table 3:** Comparison of nucleotide percent divergence and similarity among *O. brevibarba* and 8 other *Onychostoma* species

Species	Length (bp)	Nucleotide composition (%)				Similarity (%)	GenBank number
		A	T	C	G		
*O. brevibarba*	16,602	31.38	25.1	27.44	16.08		MG523272
*O. simum*	16,601	31.31	24.3	28.28	16.11	89.4	KF021233
*O. barbatulum*	16,597	31.45	25.15	27.40	16.00	91.3	KC896762
*O. barbatum*	16,589	31.54	24.49	28.05	15.91	90.7	KT438512
*O. alticorpus*	16,604	30.91	23.55	29.01	16.53	89.3	NC_021473
*O. rara*	16,590	31.49	24.15	28.47	15.88	89.6	KF626377
*O. gerlachi*	16,597	31.38	24.24	28.29	16.09	89.5	KP244449
*O. macrolepis*	16,595	31.29	24.53	27.79	16.21	90.5	KF999680
*O. lini*	16,595	31.62	24.57	27.94	15.87	90.8	JQ343982

**Fig. 3:**
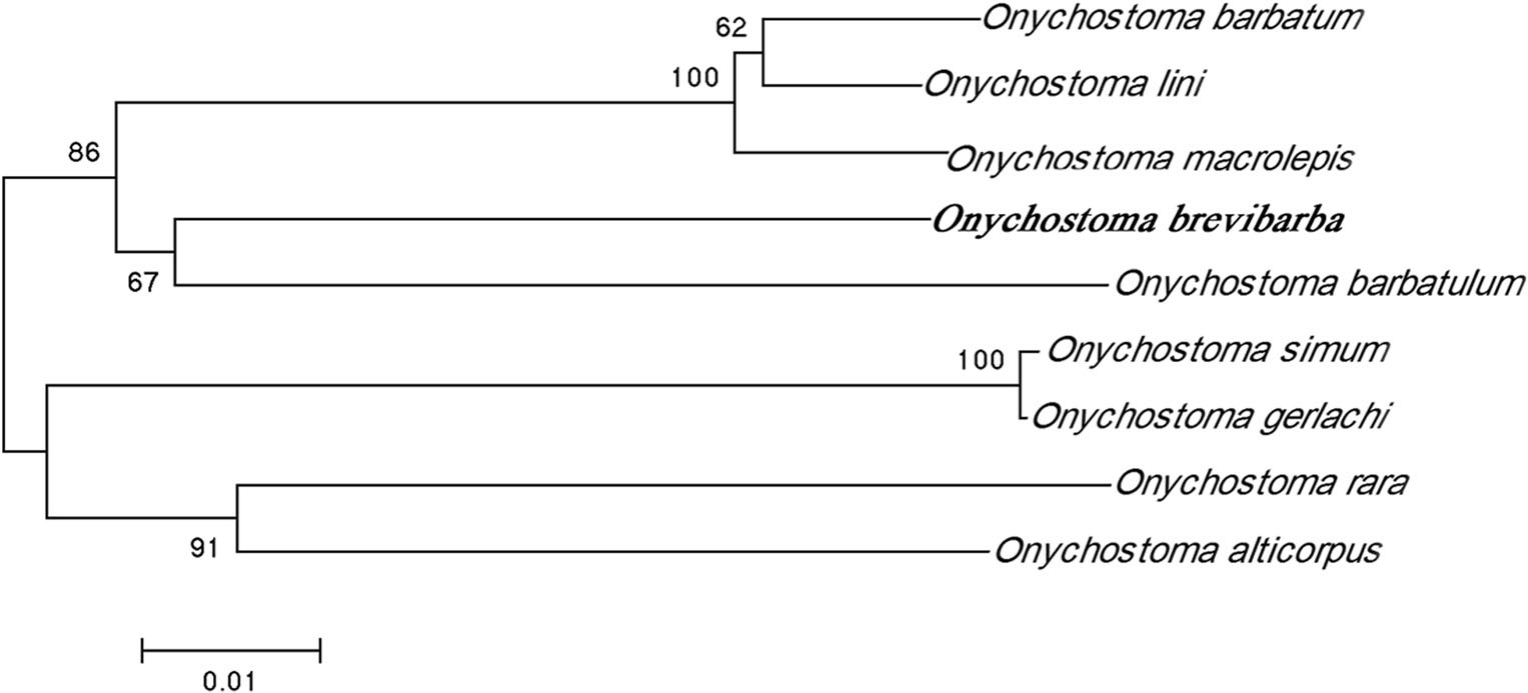
The neighbor-joining evolutionary tree reconstructed based on complete mitochondrial genomic sequences from nine *Onychostoma species* from NCBI GenBank (with accession numbers): *O. simum* (KF021233), *O. barbatulum* (KC896762), *O. barbatum* (KT438512), *O. alticorpus* (NC_021473), *O. rara* (KF626377), *O. gerlachi* (KP244449), *O. macrolepis* (KF999680), and *O. lini* (JQ343982). The scale bar indicates the nucleotide diversity between sequences, and the bootstrapping value of each branch is shown in front of the node

The phylogenetic tree was created by bootstrapping and neighbor joining with the complete mtDNA sequences (Fig. 3). Phylogenetic analysis indicated that *O. brevibarba* is most closely related to *O. barbatulum*, only differing by 8.7%.

### Overview of the PacBio Sequencing Datasets

Using equally pooled RNAs extracted from five different tissues of *O. barbatulum*, one library with cDNA insert sizes 0.5–6 kb was prepared. The libraries were sequenced using two SMRT cells on the PacBio Sequel platform. The two cells generated 1,365,887 polymerase reads, among which 792,917 ROIs were successfully extracted with mean lengths of 1900 bp and 11.40 passes (Table 4).

**Table 4:** Summary of reads of insert

Movie	Library size	Reads number Of insert	Reads bases of insert	Mean reads length of insert	Mean number of passes
m54136	0.5–6 K	408,733	795,808,888	1947.01	13.2
m54065	0.5–6 K	384,184	713,245,218	1856.52	9.47

Depending on whether 5′/3′ primer sequences and poly A tails were detected, totally, 585,750 FL non-chimeric transcripts were further extracted from the ROIs of the library. After clustering using smrtlink5.1, 259,042 non-redundant isoform-level transcripts were produced, including 172,126 mRNAs and 86,916 lncRNAs. The average length of mRNA was 2176.36 bp (Supplementary Table 3). Finally, a total of 120,239 unigene sequences were obtained.

### Functional Annotation of the *O. barbatulum* Transcriptome

**Fig. 4:**
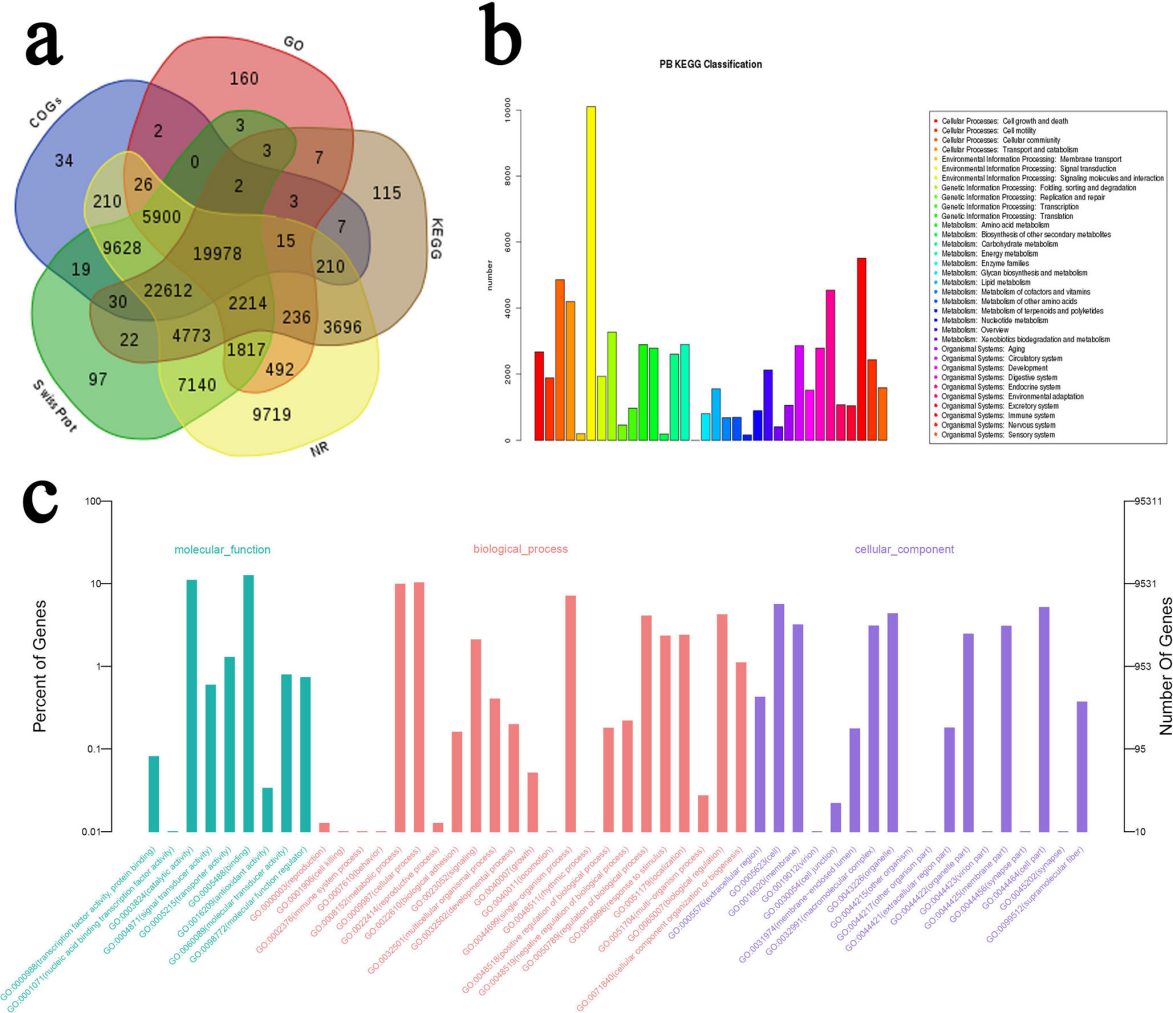
Functional annotation of the *O. brevibarba* full-length transcriptome. **a** Venn diagram of NR, GO, COG, KEGG, and Swiss-Prot annotation results. **b** Classification of KEGG annotation. **c** Classification of GO annotation

To analyze the function of the 120,239 unigenes, we used five databases, including NR, Swiss-Prot, COG, GO, and KEGG, to perform functional annotations. A total of 91,542 unigenes (76.13%) were successfully matched to known sequences in at least one of the five databases, and 19,978 unigenes were found to have high-confidence homologs in all five databases (Fig. 4a and Table 5). A total of 73,718 unigenes were successfully matched KEGG pathway; among these unigenes, as many as 12,236 unigenes were assigned to different environmental information processing, including 10,102 “signal transduction,” 1932 “signaling molecules and interaction,” and 202 “membrane transport” (Fig. 4b and Supplementary Table 4). For COG annotation, a total of 67,296 unigenes were assigned to 51 functional categories. The majority of annotated unigenes were assigned to “protein binding,” “ATP binding,” “integral component of membrane,” “membrane,” and “oxidation-reduction process” (Fig. 4c and Supplementary Table 5).

**Table 5:** Statistics of annotation

Database	COG	KEGG	NR	Swiss-Prot	GO	Refseq	Total annotated
Gene number	58,675	53,922	88,665	74,237	30,857	90,495	91,542
Annotation ratio (%)	48.8	44.85	73.74	61.74	25.66	75.26	76.13

### Widespread Distribution of mRNA Isoforms

**Fig. 5:**
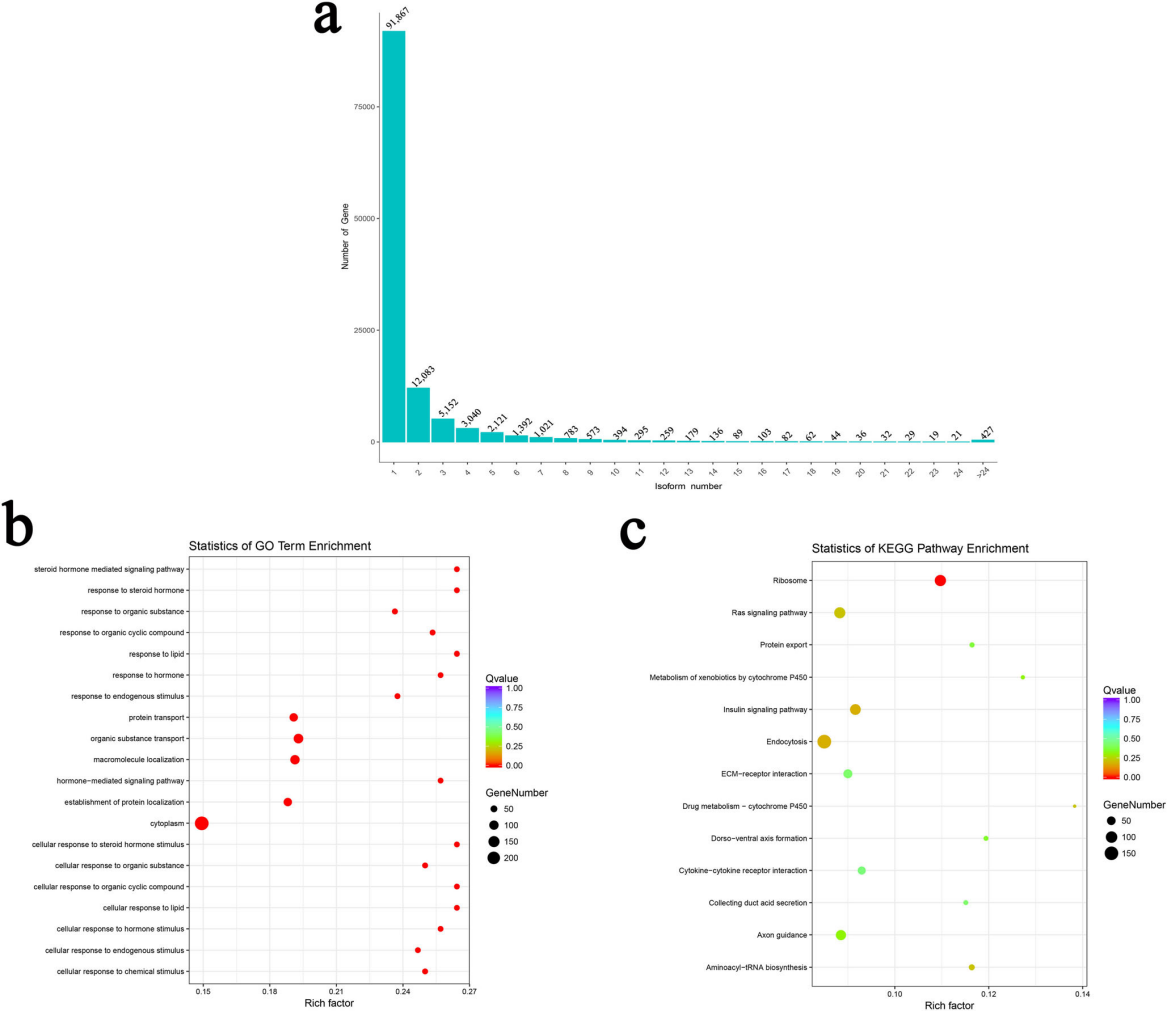
Alternative isoform analysis of the *O. brevibarba* full-length transcriptome. **a** Distribution of unigenes that have one or more alternative isoforms. **b** The top 5% alternative isoform numbers of unigenes GO enrichment. **c** The top 5% alternative isoform numbers of unigenes KEGG enrichment

One of the most important features of PacBio Sequencing is the ability to identify alternative splicing (AS), alternative transcription initiation, and alternative polyadenylation by directly comparing isoforms of the same gene. Among the 120,239 unigenes identified in *O. barbatulum*, 23.60% (28,372) have two or more isoforms and 13.55% (16,289) have three or more distinct isoforms (Fig. 5a and Supplementary Table 6). Functional enrichment analysis was performed on the top 5% AS unigenes. The GO analysis of these AS unigenes mainly involved cellular response (Fig. 5b). The KEGG pathway annotation enabled us to assign 693 unigenes to 13 pathways. Pathway enrichment analysis revealed that the top 5 enriched pathways were endocytosis, ribosome, ras signaling pathway, insulin signaling pathway, and axon guidance (Fig. 5c).

**Fig. 6:**
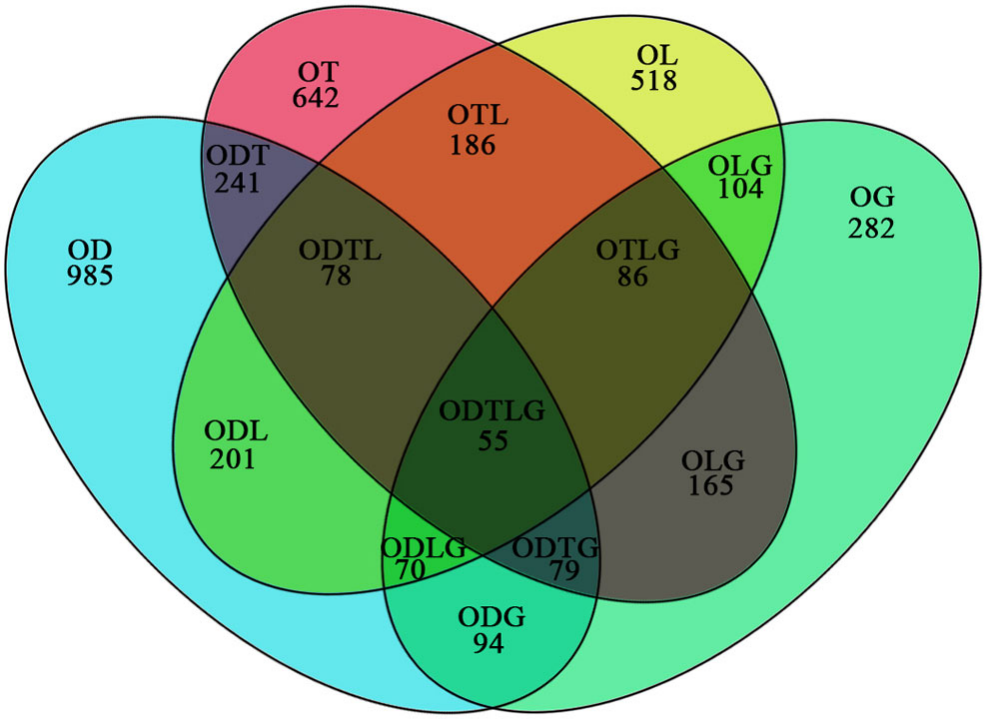
Venn diagram of shared AS variants among *O. brevibarba* and four teleost species. For shared regions, O is *O. brevibarba*, D *Danio rerio*, T *Takifugu rubripes*, L *Oryzias latipes*, G *Gasterosteus aculeatus*. OD represents the number of AS variants shared only between *O. brevibarba* and *Danio rerio*

A total of 2452 AS transcripts in *O. barbatulum* were shared with at least one of the four teleost species and varied from 40.17% (985) in *Danio rerio* to 11.5% (282) in *Gasterosteus aculeatus*. In addition, 226 AS transcripts were shared between two or more species. *O. barbatulum* and *Danio rerio* share closer evolutionary relationships than with the more distantly related *Takifugu rubripes*, *Oryzias latipes*, and *Gasterosteus aculeatus*, and shared larger numbers of AS gene identities with the three other teleost species (Fig. 6).

To validate the accuracy of the splice isoforms detected with SMRT, we randomly selected 6 genes. We performed RT-PCR using RNAs from the liver, eye, muscular, kidney, and fin ray. The gel banding pattern and the size of the fragments were consistent with the splice isoforms identified from Iso-Seq data (Supplementary Fig. 1).

## Discussion

A total of 19 valid species (subspecies) of the genus *Onychostoma* had been recognized globally (Yue et al. 2000; Yin and Dai 2014). Some of this species of *Onychostoma* were locally known to be of high economic value, even supporting a local flourishing fishery (Han et al. 2015; Song et al. 2018). However, some species of the *Onychostoma* have declined rapidly on account of the environmental damage and the over hunt by human (Yu et al. 2017). Besides, most of the previous researches on species of the genus focus on morphology and geographic distribution; studies on molecular biology and cell biology are lacking. More research is urgently needed to provide molecular and cell information for species identification and conservation of the diversity of the genus. In the present study, we analyzed the chromosomal numbers and karyotype, complete mitochondrial genome, and full-length transcriptome dataset of *O. brevibarba.* Our study could facilitate further exploration of the genomic signatures of *O. brevibarba* and provide potential insights into unveiling the evolutionary history of species of *Onychostoma*.

The chromosomal numbers and karyotype can be used as markers to analyze the ploidy levels in fishes (Liu et al. 2007), and to date, the chromosomal numbers and karyotype of 7 species of *Onychostoma* have been studied, including those of *O. simus* (Yu et al. 1987), *O. rara* (Dai et al. 2012), *O. gerlachi* (Pei-Zhen 2012), *O. lini* (Dai 2013), *O. macrolepis* (Yin and Dai 2014), *O. alticorpus* (Han et al. 2015), and *O. barbatulum* (Tseng et al. 2017). All these species are diploids, and their chromosomal numbers are 50 (Table 1). In this study, 93% of the chromosomal metaphases of all the examined samples possessed 50 chromosomes, indicating that the *O. brevibarba* specimens were diploids with 50 chromosomes (2*n* = 50) (Fig. 2a). These chromosomal numbers are consistent with those of other *Onychostoma* species. Yet, the karyotypic formula of *O. brevibarba* is 14 m + 12 sm + 8 st + 16 t, which is different from that of other *Onychostoma* species (Fig. 2b and Table 1). These results show that the different *Onychostoma* species have different karyotypes, so the karyotype could be used as the chromosomal marker to identify this species.

There have been multiple recent studies of the complete mitochondrial genome of *Onychostoma* species including *O. simum* (KF021233), *O. barbatulum* (KC896762), *O. rara* (KF626377), *O. gerlachi* (KP244449), *O. lini* (JQ343982) (Huang et al. 2012), *O. alticorpus* (NC_021473) (Cheng et al. 2014), *O. macrolepis* (KF999680), and *O. barbatum* (KT438512). Comparative analysis of the complete mtDNA sequences among *O. brevibarba* and other *Onychostoma* species has been conducted, and the results showed that the number, size, and order or arrangement of genes encoded on the mitochondrial genomes of *O. brevibarba* are similar to those typically found in other *Onychostoma* species (Tables 2 and 3). The lengths of the complete genome were all in the range of 16,589 to 16,607 bp (Table 3). The overall base composition was slightly AT rich (Table 3), and each genome contained the same 22 tRNA genes, 13 PCGs, 2 rRNA genes, and 2 main non-coding regions of the CR along with the OL. Sequence comparisons of the mitogenomes between *O. brevibarba* and other *Onychostoma* species revealed sequence similarities above 89.3% in all cases (Table 3), and the highest similarity (91.3%) was detected between *O. brevibarba* and *O. barbatula*, while the lowest (89.3%) was between *O. brevibarba* and *O. alticorpus* (Table 3). Molecular phylogeny suggests that *O. brevibarba* is most closely related to *O. barbatula*, differing by 8.7% (Fig. 2 and Table 3). These results provide further evidence that *O. brevibarba* is a new member of the genus *Onychostoma*. The complete mtDNA sequences of *O. brevibarba* provide fundamental data for further population genetics, conversation genetics, and captive breeding studies on this cyprinid fish species. These data will contribute to uncovering the related generic groups and the genetic and evolutionary relationships among *Onychostoma* in the *Cypriniformes* in different areas of mainland China and Asia.

DNA sequencing technology has been promoting the development of life science. Recently, single-molecule long-read sequencing technology from Pacific Biosciences (PacBio) has provided an efficient approach to sequence full-length cDNA molecules, which has been successively used for whole-transcriptome profiling in some animal and plant species (Zhang et al. 2018a; Yi et al. 2018; Zhang et al. 2018b). In this study, the full-length transcriptome of *O. brevibarba* was firstly sequenced with using the PacBio SMRT technique and comprehensively analyzed. We aim to provide a large amount of gene information for *Onychostoma* that lack genomic data. Our result demonstrated that full-length RNA sequencing is an efficient technique for obtaining reliable full-length transcript sequence information in *O. brevibarba*. The full-length transcriptome dataset of *O. brevibarba* comprised 120,239 unigenes (Fig. 4 and Supplementary Table 3). Among the unigenes, 91,542 were functionally annotated. *O. brevibarba* inhabit in hill stream, with clear water, slow-flowing, and mixed substrate (Song et al. 2018). In this study, KEGG pathway analyses revealed that environmental information processing including signal transduction and signaling molecules and interaction are specifically enriched in *O. brevibarba* (Fig. 4b and Supplementary Table 4)*.* These results may explain the possible reason for *O. brevibarba* in clear water.

AS plays important roles in post-transcriptional regulation in eukaryotes. Although AS profiles have been well studied in higher vertebrate, they have not been well studied in fish (Tan et al. 2018b). Previous studies showed that AS was highly regulated to adapt to environmental stimuli in eukaryotes (Tan et al. 2018a, b; Fujikake et al. 2005). In this study, 26,794 (22.28%) unigenes were found to have two or more isoforms (Fig. 5 and Supplementary Table 6). Functional enrichment of top 5% AS unigenes showed that these unigenes are involved in cellular response (Fig. 5b). Our results suggest that AS may be a general mechanism of fish to adapt to the environment. A comparison between the *O. brevibarba* and four teleost species analyzed by Lu et al. (2010) showed that AS were widespread in teleost. In addition, the degree of conservation of AS between *O. brevibarba* and *Danio rerio* was the highest. This result indicated that closer evolutionary relationships shared larger numbers of AS gene identities (Lu et al. 2010; Kijewska et al. 2018).

In summary, this is the firstly reported chromosomal numbers, karyotype, complete mitochondrial genome, and full-length transcriptome of *O. brevibarba*. Our study provided many new insights into cytology and molecular characteristics of *O. brevibarba*; it laid the foundation for further research, utilization, and protection of this species.

## Electronic supplementary material

ESM 1 (DOCX 139 kb)

ESM 2 (XLSX 10 kb)

ESM 3 (XLSX 11 kb)

ESM 4 (XLSX 125754 kb)

ESM 5 (XLSX 1322 kb)

ESM 6 (XLSX 1039 kb)

ESM 7 (XLSX 1954 kb)
