# Supplementary material for: Analysis of Chromosomal Numbers, Mitochondrial Genome, and Full-Length Transcriptome of Onychostoma brevibarba
Source: Mar Biotechnol (NY). 2019 Jun 15;21(4):515–25. doi: 10.1007/s10126-019-09899-6 (PMC6679832; doi:10.1007/s10126-019-09899-6)
Supplement: Supplementary file 1 — (DOCX 139 kb) [file 10126_2019_9899_MOESM1_ESM.docx]

Analysis of chromosomal numbers, mitochondrial genome and full-length transcriptome in *Onychostoma brevibarba* reveal their genetic signatures

Fangzhou Hu^ab1^, Jingjing Fan ^a1^, Chang Wu ^a1^, Ming Zhu^a^, Yunfan Zhou^a^, Shi Wang^a^, Chun Zhang^a^, Min Tao^a^, Rurong Zhao^a^, Chenchen Tang^a^, Kaikun Luo^a^, Qinbo Qin^a^, Ming Ma^b^, Bo Chen^b^, Shaojun Liu ^a*^

^a^State Key Laboratory of Developmental Biology of Freshwater Fish, College of Life Sciences, Hunan Normal University，Changsha, 410081, Hunan, P. R. of China

^b^Key Laboratory of Phytochemical R&D of Hunan Province, Key Laboratory of Chemical Biology & Traditional Chinese Medicine Research, Ministry of Education, Hunan Normal University, Changsha 410081, China.

^1^These authors contributed equally to this work.

^*^Correspondence and requests for materials should be addressed to S.L. (email: lsj@hunnu.edu.cn).


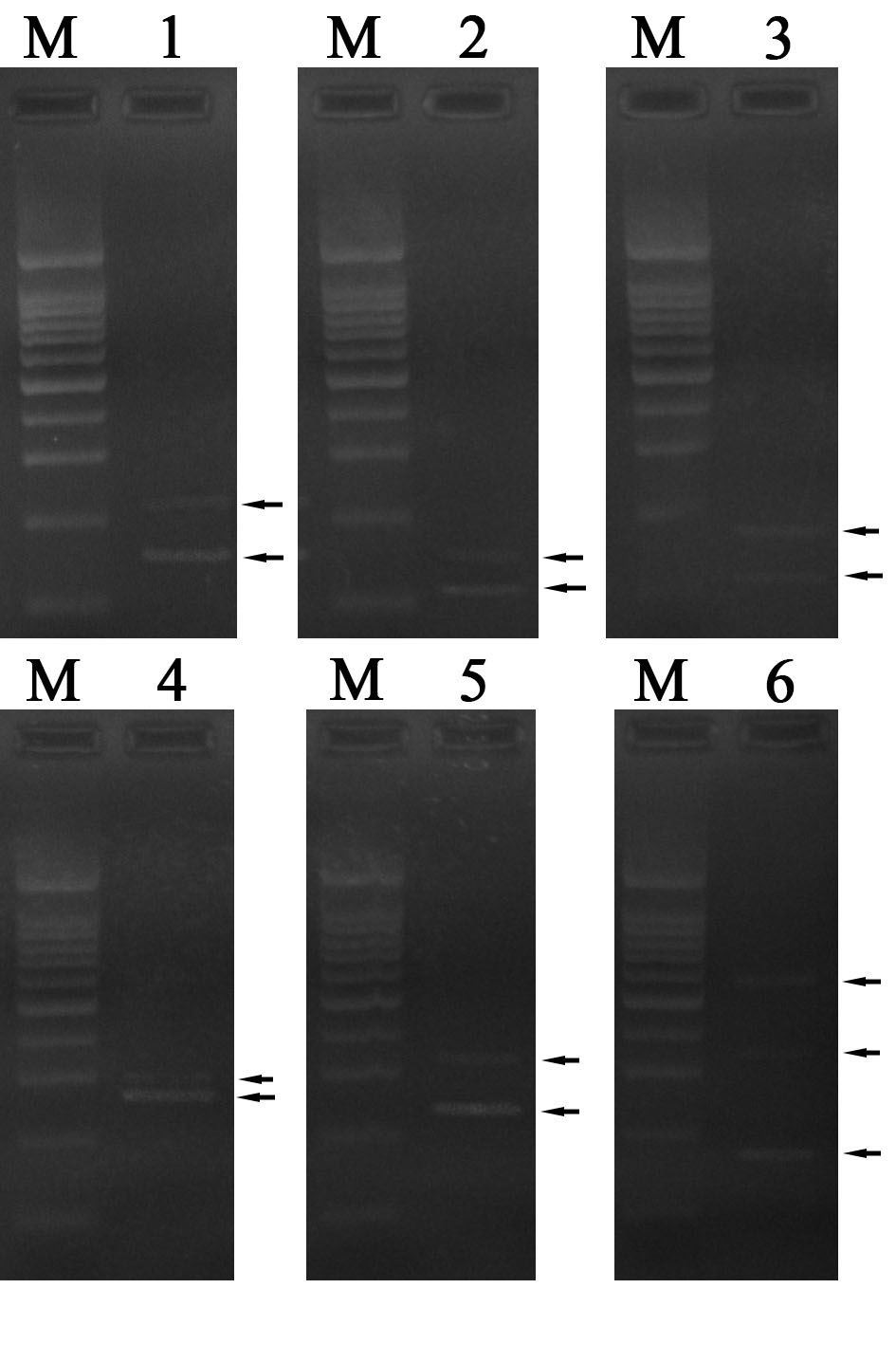


Supplementary Figure 1 RT-PCR validation of AS events and isoforms in 6 candidate genes. Arrows, PCR products; M, DNA 100 bp ladder; 1, PB.2068; 2, PB.6640; 3, PB.8151; 4, PB.7072; 5, PB4006; 6, PB.7104.
